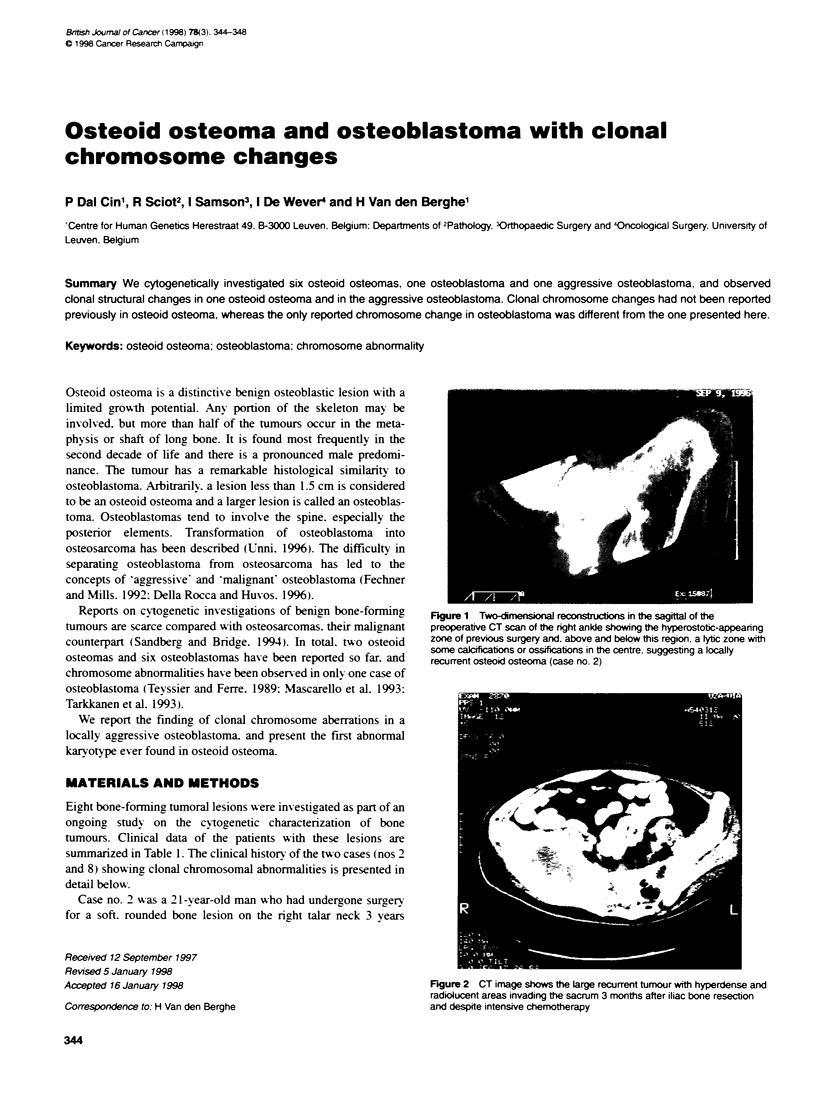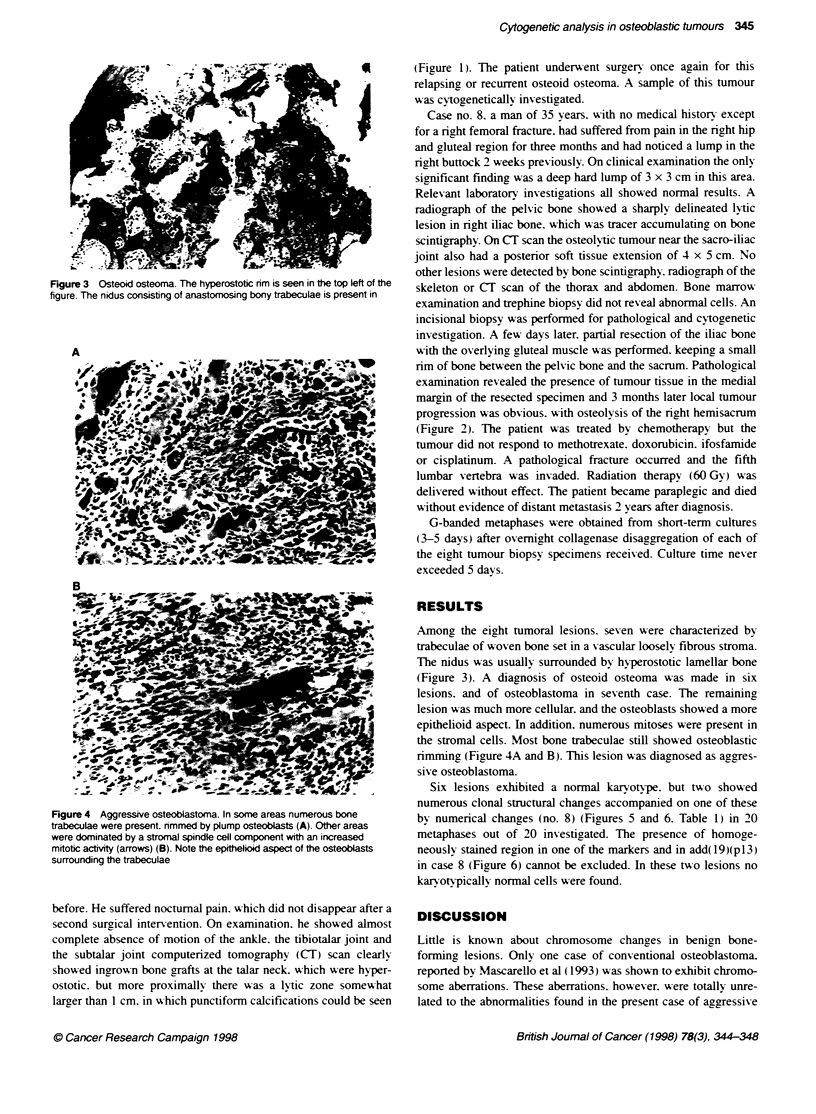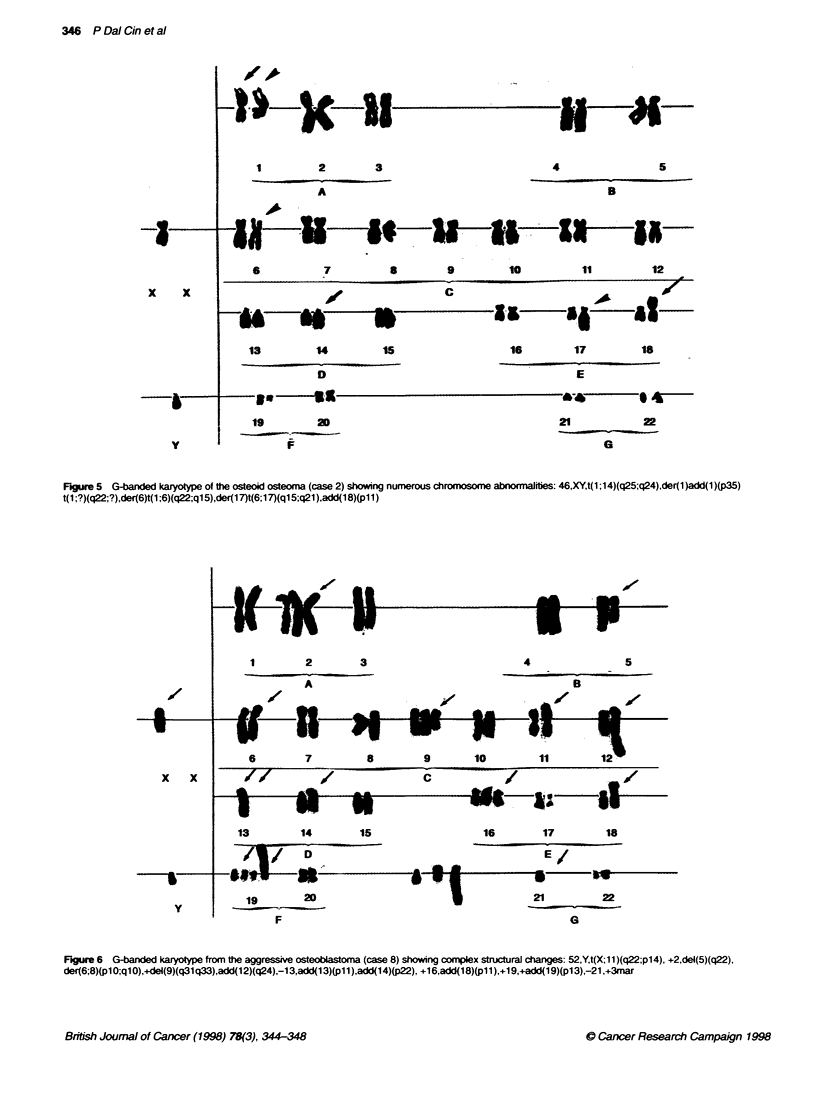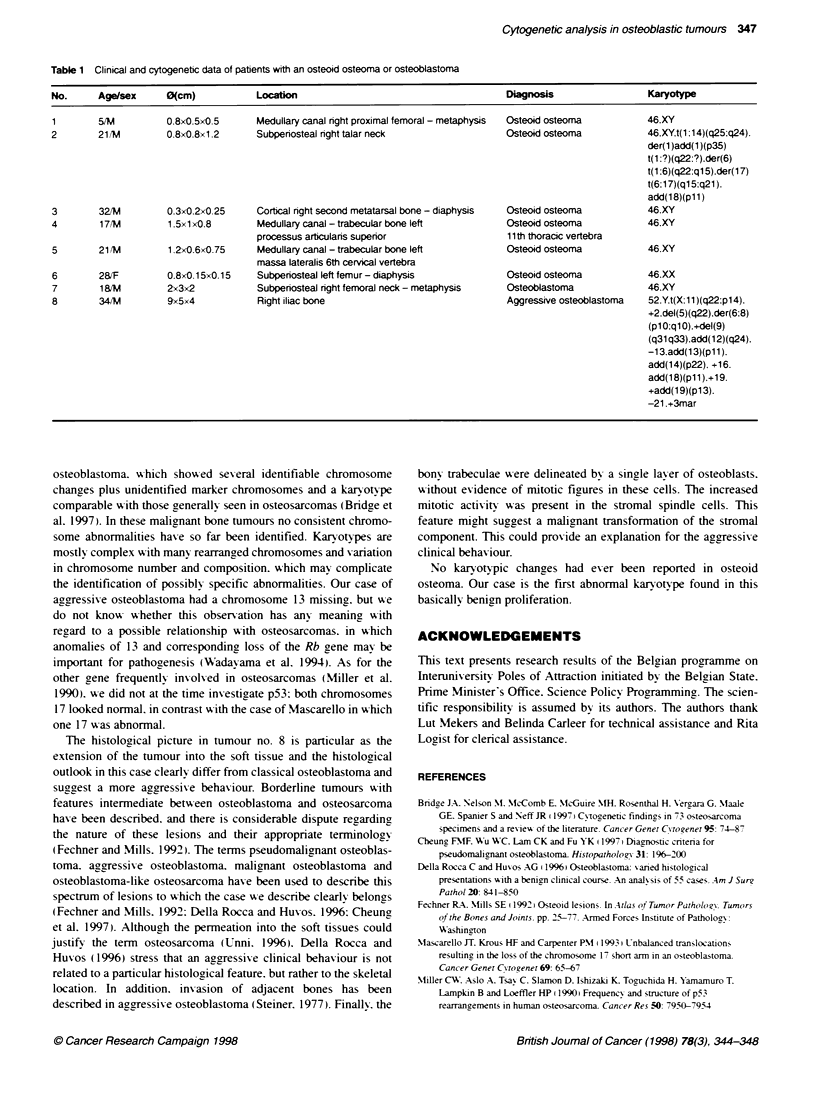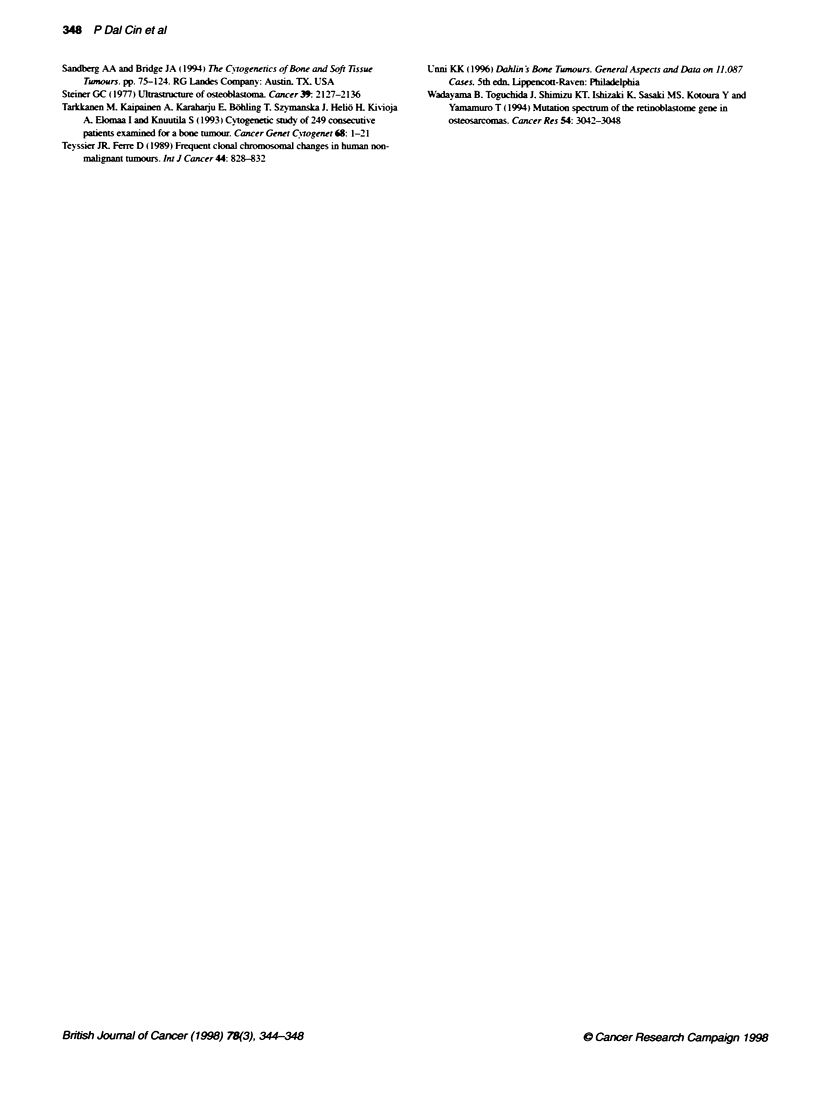# Osteoid osteoma and osteoblastoma with clonal chromosome changes.

**DOI:** 10.1038/bjc.1998.496

**Published:** 1998-08

**Authors:** P. Dal Cin, R. Sciot, I. Samson, I. De Wever, H. Van den Berghe

**Affiliations:** Centre for Human Genetics, Leuven, Belgium.

## Abstract

**Images:**


*British Journal of Cancer* (1998) **78**(3). 344–348
© 1998 Cancer Research Campaign

### Osteoid osteoma and osteoblastoma with clonal chromosome changes

**P Dal Cin[1], R Sciot[2], I Samson[3], I De Wever[4] and H Van den Berghe[1]**

[1]Centre for Human Genetics Herestraat 49. B-3000 Leuven. Belgium: Departments of [2]Pathology. [3]Orthopaedic Surgery and [4]Oncological Surgery. University of Leuven. Belgium

**Summary** We cytogenetically investigated six osteoid osteomas, one osteoblastoma and one aggressive osteoblastoma, and observed clonal structural changes in one osteoid osteoma and in the aggressive osteoblastoma. Clonal chromosome changes had not been reported previously in osteoid osteoma, whereas the only reported chromosome change in osteoblastoma was different from the one presented here.

**Keywords:** osteoid osteoma; osteoblastoma; chromosome abnormality

Osteoid osteoma is a distinctive benign osteoblastic lesion with a limited growth potential. Any portion of the skeleton may be involved. but more than half of the tumours occur in the metaphysis or shaft of long bone. It is found most frequently in the second decade of life and there is a pronounced male predominance. The tumour has a remarkable histological similarity to osteoblastoma. Arbitrarily. a lesion less than 1.5 cm is considered to be an osteoid osteoma and a larger lesion is called an osteoblastoma. Osteoblastomas tend to involve the spine. especially the posterior elements. Transformation of osteoblastoma into osteosarcoma has been described (Unni. 1996). The difficulty in separating osteoblastoma from osteosarcoma has led to the concepts of 'aggressive' and 'malignant' osteoblastoma (Fechner and Mills. 1992: Della Rocca and Huvos. 1996).

Reports on cytogenetic investigations of benign bone-forming tumours are scarce compared with osteosarcomas. their malignant counterpart (Sandberg and Bridge. 1994). In total. two osteoid osteomas and six osteoblastomas have been reported so far. and chromosome abnormalities have been observed in only one case of osteoblastoma (Teyssier and Ferre. 1989: Mascarello et al. 1993: Tarkkanen et al. 1993).

We report the finding of clonal chromosome aberrations in a locally aggressive osteoblastoma. and present the first abnormal karyotype ever found in osteoid osteoma.

### MATERIALS AND METHODS

Eight bone-forming tumoral lesions were investigated as part of an ongoing study on the cytogenetic characterization of bone tumours. Clinical data of the patients with these lesions are summarized in Table 1. The clinical history of the two cases (nos 2 and 8) showing clonal chromosomal abnormalities is presented in detail below.

Case no. 2 was a 21-year-old man who had undergone surgery for a soft. rounded bone lesion on the right talar neck 3 years

**Figure 1** Two-dimensional reconstructions in the sagittal of the preoperative CT scan of the right ankle showing the hyperostotic-appearing zone of previous surgery and. above and below this region. a lytic zone with some calcifications or ossifications in the centre. suggesting a locally recurrent osteoid osteoma (case no. 2)

**Figure 2** CT image shows the large recurrent tumour with hyperdense and radiolucent areas invading the sacrum 3 months after iliac bone resection and despite intensive chemotherapy

*Received 12 September 1997*
*Revised 5 January 1998*
*Accepted 16 January 1998*

*Correspondence to:* H Van den Berghe

**Figure 3** Osteoid osteoma. The hyperostotic rim is seen in the top left of the figure. The nidus consisting of anastomosing bony trabeculae is present in

A

B

**Figure 4** Aggressive osteoblastoma. In some areas numerous bone trabeculae were present, rimmed by plump osteoblasts (**A**). Other areas were dominated by a stromal spindle cell component with an increased mitotic activity (arrows) (**B**). Note the epithelioid aspect of the osteoblasts surrounding the trabeculae before. He suffered nocturnal pain, which did not disappear after a second surgical intervention. On examination, he showed almost complete absence of motion of the ankle, the tibiotalar joint and the subtalar joint computerized tomography (CT) scan clearly showed ingrown bone grafts at the talar neck, which were hyperostotic, but more proximally there was a lytic zone somewhat larger than 1 cm, in which punctiform calcifications could be seen (Figure 1). The patient underwent surgery once again for this relapsing or recurrent osteoid osteoma. A sample of this tumour was cytogenetically investigated.

Case no. 8, a man of 35 years, with no medical history except for a right femoral fracture, had suffered from pain in the right hip and gluteal region for three months and had noticed a lump in the right buttock 2 weeks previously. On clinical examination the only significant finding was a deep hard lump of 3 × 3 cm in this area. Relevant laboratory investigations all showed normal results. A radiograph of the pelvic bone showed a sharply delineated lytic lesion in right iliac bone, which was tracer accumulating on bone scintigraphy. On CT scan the osteolytic tumour near the sacro-iliac joint also had a posterior soft tissue extension of 4 × 5 cm. No other lesions were detected by bone scintigraphy, radiograph of the skeleton or CT scan of the thorax and abdomen. Bone marrow examination and trephine biopsy did not reveal abnormal cells. An incisional biopsy was performed for pathological and cytogenetic investigation. A few days later, partial resection of the iliac bone with the overlying gluteal muscle was performed, keeping a small rim of bone between the pelvic bone and the sacrum. Pathological examination revealed the presence of tumour tissue in the medial margin of the resected specimen and 3 months later local tumour progression was obvious, with osteolysis of the right hemisacrum (Figure 2). The patient was treated by chemotherapy but the tumour did not respond to methotrexate, doxorubicin, ifosfamide or cisplatinum. A pathological fracture occurred and the fifth lumbar vertebra was invaded. Radiation therapy (60 Gy) was delivered without effect. The patient became paraplegic and died without evidence of distant metastasis 2 years after diagnosis.

G-banded metaphases were obtained from short-term cultures (3–5 days) after overnight collagenase disaggregation of each of the eight tumour biopsy specimens received. Culture time never exceeded 5 days.

### RESULTS

Among the eight tumoral lesions, seven were characterized by trabeculae of woven bone set in a vascular loosely fibrous stroma. The nidus was usually surrounded by hyperostotic lamellar bone (Figure 3). A diagnosis of osteoid osteoma was made in six lesions, and of osteoblastoma in seventh case. The remaining lesion was much more cellular, and the osteoblasts showed a more epithelioid aspect. In addition, numerous mitoses were present in the stromal cells. Most bone trabeculae still showed osteoblastic rimming (Figure 4A and B). This lesion was diagnosed as aggressive osteoblastoma.

Six lesions exhibited a normal karyotype, but two showed numerous clonal structural changes accompanied on one of these by numerical changes (no. 8) (Figures 5 and 6, Table 1) in 20 metaphases out of 20 investigated. The presence of homogeneously stained region in one of the markers and in add(19)(p13) in case 8 (Figure 6) cannot be excluded. In these two lesions no karyotypically normal cells were found.

### DISCUSSION

Little is known about chromosome changes in benign bone-forming lesions. Only one case of conventional osteoblastoma, reported by Mascarello et al (1993) was shown to exhibit chromosome aberrations. These aberrations, however, were totally unrelated to the abnormalities found in the present case of aggressive

© Cancer Research Campaign 1998

**Figure 5** G-banded karyotype of the osteoid osteoma (case 2) showing numerous chromosome abnormalities: 46,XY,t(1;14)(q25;q24),der(1)add(1)(p35) t(1;?)(q22;?),der(6)t(1;6)(q22;q15),der(17)t(6;17)(q15;q21),add(18)(p11)

**Figure 6** G-banded karyotype from the aggressive osteoblastoma (case 8) showing complex structural changes: 52,Y,t(X;11)(q22;p14), +2,del(5)(q22), der(6;8)(p10;q10),+del(9)(q31q33),add(12)(q24),–13,add(13)(p11),add(14)(p22), +16,add(18)(p11),+19,+add(19)(p13),–21,+3mar

**Table 1** Clinical and cytogenetic data of patients with an osteoid osteoma or osteoblastoma

| No. | Age/sex | Ø(cm) | Location | Diagnosis | Karyotype |
|---|---|---|---|---|---|
| 1 | 5/M | 0.8×0.5×0.5 | Medullary canal right proximal femoral – metaphysis | Osteoid osteoma | 46.XY |
| 2 | 21/M | 0.8×0.8×1.2 | Subperiosteal right talar neck | Osteoid osteoma | 46.XY.t(1:14)(q25:q24). der(1)add(1)(p35) t(1:?)(q22:?).der(6) t(1:6)(q22:q15).der(17) t(6:17)(q15:q21). add(18)(p11) |
| 3 | 32/M | 0.3×0.2×0.25 | Cortical right second metatarsal bone – diaphysis | Osteoid osteoma | 46.XY |
| 4 | 17/M | 1.5×1×0.8 | Medullary canal – trabecular bone left processus articularis superior | Osteoid osteoma 11th thoracic vertebra | 46.XY |
| 5 | 21/M | 1.2×0.6×0.75 | Medullary canal – trabecular bone left massa lateralis 6th cervical vertebra | Osteoid osteoma | 46.XY |
| 6 | 28/F | 0.8×0.15×0.15 | Subperiosteal left femur – diaphysis | Osteoid osteoma | 46.XX |
| 7 | 18/M | 2×3×2 | Subperiosteal right femoral neck – metaphysis | Osteoblastoma | 46.XY |
| 8 | 34/M | 9×5×4 | Right iliac bone | Aggressive osteoblastoma | 52.Y.t(X:11)(q22:p14). +2.del(5)(q22).der(6:8) (p10:q10).+del(9) (q31q33).add(12)(q24). −13.add(13)(p11). add(14)(p22). +16. add(18)(p11).+19. +add(19)(p13). −21.+3mar |

osteoblastoma. which showed several identifiable chromosome changes plus unidentified marker chromosomes and a karyotype comparable with those generally seen in osteosarcomas (Bridge et al. 1997). In these malignant bone tumours no consistent chromosome abnormalities have so far been identified. Karyotypes are mostly complex with many rearranged chromosomes and variation in chromosome number and composition. which may complicate the identification of possibly specific abnormalities. Our case of aggressive osteoblastoma had a chromosome 13 missing. but we do not know whether this observation has any meaning with regard to a possible relationship with osteosarcomas. in which anomalies of 13 and corresponding loss of the *Rb* gene may be important for pathogenesis (Wadayama et al. 1994). As for the other gene frequently involved in osteosarcomas (Miller et al. 1990). we did not at the time investigate p53: both chromosomes 17 looked normal. in contrast with the case of Mascarello in which one 17 was abnormal.

The histological picture in tumour no. 8 is particular as the extension of the tumour into the soft tissue and the histological outlook in this case clearly differ from classical osteoblastoma and suggest a more aggressive behaviour. Borderline tumours with features intermediate between osteoblastoma and osteosarcoma have been described. and there is considerable dispute regarding the nature of these lesions and their appropriate terminology (Fechner and Mills. 1992). The terms pseudomalignant osteoblastoma. aggressive osteoblastoma. malignant osteoblastoma and osteoblastoma-like osteosarcoma have been used to describe this spectrum of lesions to which the case we describe clearly belongs (Fechner and Mills. 1992: Della Rocca and Huvos. 1996: Cheung et al. 1997). Although the permeation into the soft tissues could justify the term osteosarcoma (Unni. 1996). Della Rocca and Huvos (1996) stress that an aggressive clinical behaviour is not related to a particular histological feature. but rather to the skeletal location. In addition. invasion of adjacent bones has been described in aggressive osteoblastoma (Steiner. 1977). Finally. the bony trabeculae were delineated by a single layer of osteoblasts. without evidence of mitotic figures in these cells. The increased mitotic activity was present in the stromal spindle cells. This feature might suggest a malignant transformation of the stromal component. This could provide an explanation for the aggressive clinical behaviour.

No karyotypic changes had ever been reported in osteoid osteoma. Our case is the first abnormal karyotype found in this basically benign proliferation.

### ACKNOWLEDGEMENTS

This text presents research results of the Belgian programme on Interuniversity Poles of Attraction initiated by the Belgian State. Prime Minister's Office. Science Policy Programming. The scientific responsibility is assumed by its authors. The authors thank Lut Mekers and Belinda Carleer for technical assistance and Rita Logist for clerical assistance.

### REFERENCES

Bridge JA. Nelson M. McComb E. McGuire MH. Rosenthal H. Vergara G. Maale GE. Spanier S and Neff JR (1997) Cytogenetic findings in 73 osteosarcoma specimens and a review of the literature. *Cancer Genet Cytogenet* **95**: 74–87

Cheung FMF. Wu WC. Lam CK and Fu YK (1997) Diagnostic criteria for pseudomalignant osteoblastoma. *Histopathology* **31**: 196–200

Della Rocca C and Huvos AG (1996) Osteoblastoma: varied histological presentations with a benign clinical course. An analysis of 55 cases. *Am J Surg Pathol* **20**: 841–850

Fechner RA. Mills SE (1992) Osteoid lesions. In *Atlas of Tumor Pathology. Tumors of the Bones and Joints*. pp. 25–77. Armed Forces Institute of Pathology: Washington

Mascarello JT. Krous HF and Carpenter PM (1993) Unbalanced translocations resulting in the loss of the chromosome 17 short arm in an osteoblastoma. *Cancer Genet Cytogenet* **69**: 65–67

Miller CW. Aslo A. Tsay C. Slamon D. Ishizaki K. Toguchida H. Yamamuro T. Lampkin B and Loeffler HP (1990) Frequency and structure of p53 rearrangements in human osteosarcoma. *Cancer Res* **50**: 7950–7954

Sandberg AA and Bridge JA (1994) *The Cytogenetics of Bone and Soft Tissue Tumours*. pp. 75–124. RG Landes Company: Austin, TX, USA

Steiner GC (1977) Ultrastructure of osteoblastoma. *Cancer* **39**: 2127–2136

Tarkkanen M, Kaipainen A, Karaharju E, Böhling T, Szymanska J, Heliö H, Kivioja A, Elomaa I and Knuutila S (1993) Cytogenetic study of 249 consecutive patients examined for a bone tumour. *Cancer Genet Cytogenet* **68**: 1–21

Teyssier JR, Ferre D (1989) Frequent clonal chromosomal changes in human non-malignant tumours. *Int J Cancer* **44**: 828–832

Unni KK (1996) *Dahlin's Bone Tumours. General Aspects and Data on 11,087 Cases*. 5th edn, Lippencott-Raven: Philadelphia

Wadayama B, Toguchida J, Shimizu KT, Ishizaki K, Sasaki MS, Kotoura Y and Yamamuro T (1994) Mutation spectrum of the retinoblastome gene in osteosarcomas. *Cancer Res* **54**: 3042–3048

© Cancer Research Campaign 1998